# Candida Bezoar of the Bladder Resulting in Obstructive Uropathy: A Case Report

**DOI:** 10.7759/cureus.35691

**Published:** 2023-03-02

**Authors:** Nicholas M McGhee, Fizah Chaudhary, Muhammad Nadeem Yousaf

**Affiliations:** 1 Department of Internal Medicine, University of Missouri School of Medicine, Columbia, USA

**Keywords:** hydronephrosis, urinary tract obstruction, diabetes, immunocompromised, candida bezoars

## Abstract

*Candida* bezoar is a rare pathologic entity characterized by the colonization of a cavity by an aggregate or mass of mycelia due to local or systemic infections with *Candida* spp. *Candida* bezoar is commonly seen in immunocompromised individuals and can often present in the context of symptomatic urinary tract infection or urosepsis. The implicated risk factors for the development of *Candida* bezoars are anatomical urinary tract abnormalities, diabetes mellitus, indwelling urinary catheters, increased use of broad-spectrum antibiotics, and corticosteroids. Early clinical suspicion is essential for diagnosis to prevent the dissemination of disease and for a favorable prognosis. We report a case of a 49-year-old diabetic male who presents with hematuria, abnormal urinary flow, and left-sided flank pain for four days caused by a *Candida* bezoar of the urinary bladder resulting in unilateral obstructive uropathy despite the appropriate placement of a ureteral stent. Treatment with left nephrostomy tube, oral fluconazole, and amphotericin bladder irrigation for three days was successful. The patient’s condition improved, and he was discharged on fluconazole and was recommended to follow up with urology as an outpatient.

## Introduction

*Candida* bezoars are a colonization of a cavity by an aggregate or mass of *Candida* mycelia. While candiduria can be seen in approximately 20% of hospitalized patients, the development of *Candida* bezoars is very rare [1]. *Candida *bezoars are largely seen in immunocompromised individuals, hospitalized patients with multiple comorbidities, and those with severe illnesses requiring admission to intensive care units [2]. The precise incidence and prevalence of *Candida* bezoars are unknown. However,* Candida *bezoars are commonly seen in neonatal and elderly populations [2,3]. The implicated risk factors for the development of *Candida* bezoars are anatomical urinary tract abnormalities, diabetes mellitus, indwelling urinary catheters, increased use of broad-spectrum antibiotics, and corticosteroids [3]. The clinical presentation of *Candida* bezoars usually presents in the context of symptomatic urinary tract infection or urosepsis. Bezoars are not typically symptomatic themselves until they cause urinary tract obstruction. Obstructing bezoars may cause renal colic pain and the development of post-renal kidney injury [3]. We present a case of a 49-year-old male patient with a *Candida* bezoar of the urinary bladder resulting in unilateral obstructive uropathy despite the appropriate placement of a ureteral stent.

## Case presentation

**Figure 1:**
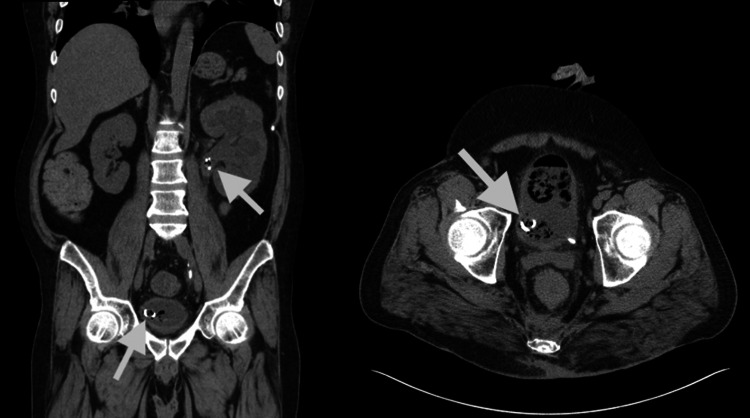
Abdominal CT scan showing left hydronephrosis with left ureteral stent in place extending from the left renal pelvis to the urinary bladder. Heterogenous filling defects containing gas noted in the urinary bladder (arrows).

A 49-year-old male with a past medical history of chronic hidradenitis suppurativa involving the axillary region bilaterally, uncontrolled type II diabetes mellitus with glycosylated hemoglobin (HbA1c) of 10.2, recurrent kidney stones, and left hydronephrosis due to ureteral stricture presented for hematuria, abnormal urinary flow, and left-sided flank pain for four days. His physical examination was significant for tenderness in the left upper quadrant, left lower quadrant, suprapubic region, and left costovertebral angle (CVA). During his initial assessment, his laboratory workup was notable for an elevated creatinine of 1.7 mg/dL (baseline: 1.1 mg/dL), mild hyperkalemia (5.3), hyperglycemia 320 mg/dL, and a leukocytosis of 12.8 k/uL. Urinalysis results were notable for glucosuria, hematuria, proteinuria with positive leukocyte esterase, and pyuria. A CT of the abdomen and pelvis demonstrated marked left hydronephrosis despite an appropriately positioned ureteral stent, a heterogenous filling defect containing gas within the urinary bladder, and an additional gas-containing filling defect present at the proximal end of the ureteral stent (Figure 1).

**Figure 2:**
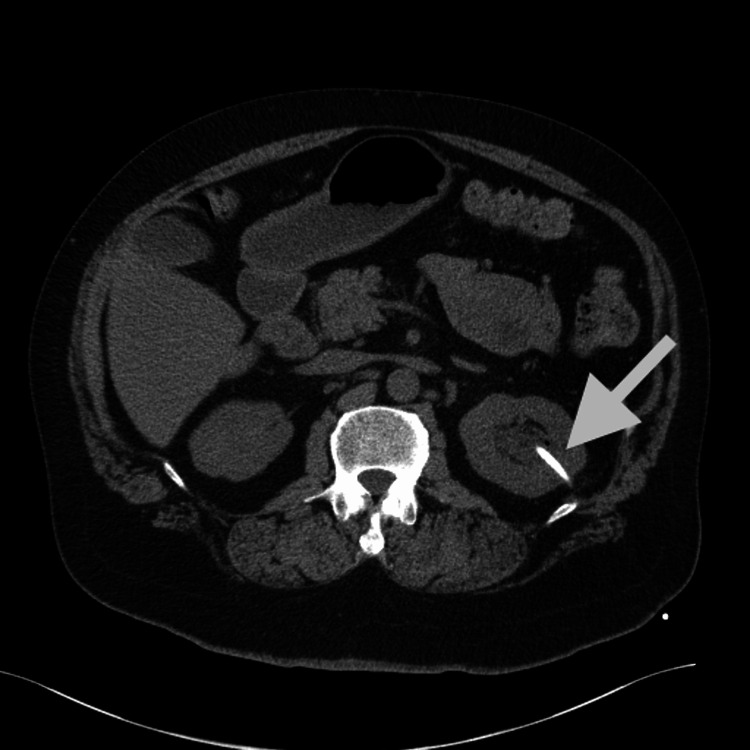
Abdominal CT scan showing the improvement of left hydronephrosis and nephrostomy tube replacing previous left ureteral stent (arrows).

Subsequently, the patient was started on intravenous piperacillin/tazobactam, and bladder irrigation was performed by urology to remove debris. Interventional radiology-guided left nephrostomy tube was placed, which drained 110 mL of purulent fluid from the ureteral collecting system. Purulent fluid later speciated *Candida albicans*. The broad-spectrum antibiotics were then switch to oral fluconazole 400 mg daily for a two-week duration, and the patient underwent amphotericin bladder irrigation for three days. Bladder irrigation resulted in the expulsion of thick tan-white fungal aggregate measuring 1.5 × 1.4 × 0.4 cm. Pathology demonstrated innumerable fungal hyphae with associated inflammation. A repeat CT scan revealed no further filling defects in the bladder or kidney (Figure 2). Blood cultures during hospitalization demonstrated no bacterial growth and were negative for fungal growth. The patient’s condition improved, and he was discharged with direction to complete the two-week course of fluconazole. The patient was recommended to follow up with urology as an outpatient. His clinical symptoms and hematuria improved on short-term follow-up. However, it is difficult to comment on long-term complications at this time. There are pending additional follow-up appointments to date.

## Discussion

This case illustrates an unusual presentation of a *Candida albicans* bezoar in the urinary bladder resulting in unilateral obstructive uropathy, with the evidence of candiduria given the positive culture for *Candida* from the urinary collecting system and multiple fungal aggregates in the bladder irrigation fluid. The risk factors for *Candida* bezoars include an immunocompromised state, increased use of antibiotics and corticosteroids, diabetes mellitus, and indwelling urinary catheters [2]. Several of these factors are present in our patient including uncontrolled type II diabetes mellitus, urinary tract abnormalities, and ureteral stenting.

*Candida albicans* is the most common fungal species causing fungal bezoars; however, other fungal isolates have been reported in the literature including other *Candida *spp., *Aspergillus *spp., *Rhizopus oryzae*, and *Geotrichum candidum* [3-5]. *Candida *bezoar infections of the bladder and upper urinary tract are a very rare phenomenon with unknown exact epidemiology. A high clinical index of suspicion with prompt medical and surgical treatment is necessary for treating *Candida* bezoar infections in immunocompromised patients. Although our case demonstrates a complication of localized *Candida* infection rather than dissemination, the mortality rate of disseminated *Candida* infections has been reported as high as 44% [6]. Diagnostic modalities include the following: ultrasonogram of the abdomen, non-contrast and contrast-enhanced abdominal CT, and MRI urogram [4].

When utilizing ultrasound, *Candida* bezoars are seen as mobile, rounded, and heterogeneously hypoechoic masses with no evidence of vascularity seen in Doppler studies. Several morphological features can be appreciated on abdominal CT/MRI scans, including rounded heterogeneous soft-tissue densities not attached to the urinary tract wall, with or without regions of gas or calcification. MRI signal characteristics of *Candida* bezoars have been rarely illustrated but include T1 scans being isointense to renal parenchyma while T2 scans being hyperintense to renal parenchyma [4]. The most described treatment approach to manage fungal bezoars is surgical drainage by nephrostomy tube and/or ureteral catheter in addition to the administration of systemic and local antifungal agents such as fluconazole or amphotericin B for a two-week duration [7]. Our patient was managed with drainage through a nephrostomy tube, a systemic antifungal treatment with fluconazole, and local antifungal therapies including bladder irrigation with amphotericin B.

## Conclusions

*Candida* bezoars are a rare entity and should be considered in the differential of immunocompromised patients presenting with candiduria. Early diagnosis and treatment are important to prevent the dissemination of disease and for a favorable prognosis.
